# Poor concordance between interferon-γ release assays and tuberculin skin tests in diagnosis of latent tuberculosis infection among HIV-infected individuals

**DOI:** 10.1186/1471-2334-9-15

**Published:** 2009-02-10

**Authors:** Naasha J Talati, Ulrich Seybold, Bianca Humphrey, Abiola Aina, Jane Tapia, Paul Weinfurter, Rachel Albalak, Henry M Blumberg

**Affiliations:** 1 Division of Infectious Diseases, Emory University, School of Medicine, Atlanta, GA, USA; 2 Division of Infectious Diseases, Medical Policlinic, Ludwig Maximilians – University, Munich, Germany; 3 Division of Tuberculosis Elimination, Centers for Disease Control and Prevention (CDC), Atlanta, GA, USA; 4 Division of Infectious Diseases, Grady Memorial Hospital, Atlanta, GA, USA

## Abstract

**Background:**

A new generation of diagnostic tests, the interferon-γ release assays (IGRAs), have been developed for the detection of latent tuberculosis infection (LTBI). Limited data are available on their use in HIV-infected persons.

**Methods:**

A cross-sectional study was carried out at 2 HIV clinics in Atlanta to assess the utility of two IGRA tests (T-SPOT.TB [TSPOT] and QuantiFERON-TB Gold in Tube [QFT-3G]) compared to the tuberculin skin test (TST).

**Results:**

336 HIV-infected persons were enrolled. Median CD4 count was 335 cells/μl and median HIV viral load was 400 copies/ml. Overall, 27 patients (8.0%) had at least 1 positive diagnostic test for LTBI: 7 (2.1%) had a positive TST; 9 (2.7%) a positive QFT-3G; and 14 (4.2%) a positive TSPOT. Agreement between the 3 diagnostic tests was poor: TST and TSPOT, [κ = 0.16, 95% CI (-0.06, 0.39)], TST and QFT-3G [κ = 0.23, 95% CI (-0.05, 0.51)], QFT-3G and TSPOT [κ = 0.06, 95% CI (-0.1, 0.2)]. An indeterminate test result occurred among 6 (1.8%) of QFT-3G and 47 (14%) of TSPOT tests. In multivariate analysis, patients with a CD4 ≤ 200 cells/μl were significantly more likely to have an indeterminate result [OR = 3.6, 95% CI (1.9, 6.8)].

**Conclusion:**

We found a low prevalence of LTBI and poor concordance between all 3 diagnostic tests. Indeterminate test results were more likely at CD4 counts ≤ 200 cells/μl. Additional studies among HIV-infected populations with a high prevalence of TB are needed to further assess the utility of IGRAs in this patient population.

## Background

The World Health Organization estimates that one third of the world's population is infected with *Mycobacterium tuberculosis*[1]. Treatment of latent tuberculosis infection (LTBI) has been emphasized as a major strategy for TB control and prevention particularly in the U.S. [2]. Those with HIV are at greatest risk of progression from infection with *M. tuberculosis *to development of active disease. In immunocompetent patients with LTBI the lifetime risk of progression to active disease is 5–10% [3]. However among patients with LTBI who have HIV co-infection the annual risk of progression is 8% [4]. Given this extremely high risk, diagnosis and treatment of LTBI among HIV-infected individuals is strongly emphasized as an important TB control strategy [4-6] and treatment of LTBI markedly reduces the risk of progression to active disease[7].

In order to enhance TB control efforts, there is an urgent need for new and better diagnostics. For more than a century the only test available to identify LTBI was the tuberculin skin test (TST). This test has a number of limitations including (1) reader variability; (2) false-positive test results due to cross-reactivity with environmental non-tuberculous mycobacteria and BCG; (3) false-negative test results due to anergy in immunosuppressed individuals; and (4) inconvenience to patients as they are required to return to get the test read [2].

In recent years, a new generation of diagnostic tests for LTBI, the T-cell based interferon-γ release assays (IGRAs), have been developed: QuantiFERON TB Gold in Tube test (QFT-3G) (Cellestis, Carnegie, Australia) and T-SPOT.TB (TSPOT) (Oxford Immunotec, Abingdon, UK) [8,9]. IGRAs measure the amount of interferon-γ released from sensitized T cells after exposure to *M. tuberculosis *antigens. These include early secreted antigenic target 6 (ESAT-6) and culture filtrate protein 10 (CFP-10), which are encoded by genes in the region of difference 1 (RD1) segment of the *M. tuberculosis *genome [9]. An additional antigen TB7.7 is included in QFT-3G. These antigens are more specific than those in the purified protein derivative (PPD) because they do not cross-react with BCG or *M. avium *complex [8]. However they do cross-react with *M. kansasii*, *M. marinum M. szulgai *and virulent strains of *M. bovis *[10]. The amount of interferon-γ released is measured using either an enzyme linked immunosorbent assay (ELISA) for QFT-3G or enzyme linked immunospot assay (ELISPOT) for TSPOT. The advantages of IGRAs over TST include better specificity, ability to perform serial tests without boosting, and logistical convenience [11].

The Centers for Disease Control and Prevention (CDC) has recommended that the currently FDA-approved whole blood IGRA (QuantiFERON-TB Gold) can be used as a diagnostic test for LTBI in place of the tuberculin skin test [12]. However, there are very limited data on the use of IGRAs among HIV-infected persons. There is only one published study that has compared both IGRAs with TST in HIV-infected individuals, and this study was limited by a small sample size [13]. CDC and others have noted that additional research is needed and recommend caution in the use of IGRAs among HIV-infected persons [6,11].

The purpose of our study was to: 1) assess the prevalence of LTBI among urban HIV-infected persons; 2) assess the utility of IGRAs, specifically TSPOT and QFT-3G, among HIV-infected persons; 3) assess the degree of concordance among three diagnostic tests for LTBI (TST, QFT-3G, and TSPOT); and 4) assess risk factors associated with indeterminate IGRA test results.

## Methods

### Study Design, Setting and Population

A cross-sectional study was carried out at 2 HIV clinics in Atlanta (Grady Health System Ponce de Leon Center and the DeKalb Board of Health) from September 2005 – July 2006. The study was approved by the Emory University Institutional Review Board (IRB) as well as IRBs at CDC and the Georgia Department of Human Resources. Patients who had positive serologic tests for HIV and were ≥ 18 years of age were eligible for enrollment into the study. All enrolled persons provided written informed consent. Demographic data, medical history, and history of BCG vaccination were obtained from medical records and patient interviews and entered on a data abstraction form. CD4 counts and HIV viral load levels were obtained from medical records and were performed within a 3-month period from the time of enrollment in the study.

### Interferon-γ Release Assays and Tuberculin Skin Testing

A trained technician who was blinded to the patients' medical history and TST results performed both IGRAs. The TSPOT test was performed as previously described and according to manufacturer's instructions [14]. Briefly, peripheral blood mononuclear cells (PBMCs) were obtained and counted to ensure that each well was plated with a minimum of 2.5 × 10^5 ^cells. Plates were incubated overnight to allow for antigen stimulation and release of interferon-γ. After incubation the wells were developed using an enzyme-conjugated secondary antibody to the interferon-γ. Colored spots were generated by the conjugated enzyme upon application of a colorimetric substrate. Spot-forming units were counted by a trained technician using a standardized magnifying lens. Spots were also counted using an AID ELISPOT Reader (ER). The technician doing the visual counting and those determining the number of spot-forming cells by ER were blinded to each other's results. The TSPOT was considered reactive if the response to either the ESAT 6 or CFP10 minus the negative control was ≥ 6 spot forming cells, or > 2 × the negative control. The result was considered indeterminate if the reading in the negative control was > 20 spot forming cells or if the reading in the positive control was < 20 spot forming cells.

For QFT-3G, 1-ml aliquots of blood were collected in three tubes, one with the negative control, one with the positive control and one with TB specific antigens. The test was performed as previously described and per the manufacturer's instructions [15]. Blood samples were centrifuged and incubated overnight. After incubation, plasma was harvested and concentrations of interferon-γ were measured by ELISA. The QFT-3G was considered positive if the interferon-γ response to TB antigens minus the negative control was ≥ 0.35 IU/ml and > 25% of the negative control; negative if these criteria were not met; and indeterminate if either the negative control had a result of > 8 IU/ml or the positive control had a result of < 0.5 IU/ml.

After blood was drawn for the IGRAs, a TST was placed using the Mantoux method [16]; 0.1 ml (5 TU) of PPD (Tubersol; Sanofi Pasteur Inc., Swiftwater, PA) was placed intradermally. The TST was measured by a trained reader between 48 and 72 hours. Induration of ≥ 5 mm was considered positive [16].

### Statistical analyses

Data were entered into an Access database (Microsoft, Redmond, WA) and analyzed using SAS (SAS Institute Inc. version 9.1, Cary, NC). Outcomes included prevalence of LTBI, concordance between tests, factors associated with a positive and indeterminate test result. Both concordance between the diagnostic tests for LTBI (IGRAs and TST) and concordance between TSPOT results by visual inspection versus an ELISPOT reader were measured. All reported TSPOT results are based on results from the ELISPOT reader. Concordance was assessed using κ, where κ >0.75 represents excellent agreement, κ values from 0.4–0.75 represent fair to good agreement and κ < 0.4 represents poor agreement, beyond chance [17]. Risk factors for test positivity were evaluated using prevalence odds ratios (OR). Due to the small number of positive test results (as a result of the low prevalence of LTBI), in most instances only univariate analysis was performed. Variables that were looked at included age, sex, race, CD4 count, HIV viral load, and selected elements of initial medical history such as history of BCG vaccination, prior history of LTBI or AIDS-defining malignancy. Prior history of LTBI was defined as a positive TST in the past. Multivariate logistic regression model was used to assess factors associated with a positive TSPOT result. The multivariate model included age, race, CD4 count and HIV viral load. A multivariate logistic regression was also used to assess factors associated with indeterminate IGRA test results. Predictors in the final model were age, sex, race and CD4 count. Correlation was also assessed between CD4 counts and interferon-γ values for QFT-3G, and CD4 counts and spot forming cells for TSPOT using Spearman's rank correlation coefficient (ρ).

## Results

### Population characteristics

A total of 336 HIV-infected persons were enrolled. The baseline characteristics of the patient population are shown in Table 1. The median CD4 count was 335 cells/μl (range 0–1380 cells/μl) and 101 (30.1%) patients had a CD4 count ≤ 200 cells/μl. Median viral load was 400 copies/ml (< 50 to > 750,000 copies/ml), and median duration of HIV diagnosis was 8 years. Two hundred thirty-one patients (69%) were on antiretroviral therapy at the time of enrollment in the study.

**Table 1:** Baseline characteristics of population, enrolled at two urban HIV clinics (n = 336).

Patient Characteristics	Numbers and Percentages
**Age**	
Mean and range	42 years (22–79 years)

**Sex**	
Male	218 (65%)
Female	118 (35%)

**Race**	
African American	286 (85.1%)
Caucasian	39 (11.6%)
Hispanic	7 (2.1%)
Asian	4 (1.2%)

**Place of Birth**	
Born outside the United States	29 (8.6%)

**HIV History**	
CD4 count, median and range	334 (0–1380 cells/μl)
Viral load, median and range	400 (< 50–> 750,000 copies/ml)
Duration of HIV diagnosis, median and range	8 (0–26 years)
Patients on HAART	231 (68.8%)
History of opportunistic infection	202 (60%)

**Past Medical History**	
Prior history of latent tuberculosis infection	7 (2.1%)
History of BCG vaccination	25 (7.4%)
Diabetes mellitus	23 (6.9%)
Chronic renal insufficiency	12 (3.6%)
History of malignancy	14 (4.2%)
Anytime smoker	186 (55.4%)
Intravenous drug use	64 (19%)

HIV = Human Immunodeficiency Virus

HAART = Highly Active Antiretroviral Therapy

BCG = Bacille Calmette-Guérin

### Diagnostic Tests for Latent Tuberculosis Infection (LTBI)

Overall, 27 (8.0%) of 336 patients had at least one positive diagnostic test for LTBI. Among the 278 patients who had their TST read, seven (2.5%) were positive; 9 of the 336 patients (2.7%) had a positive QFT-3G; and 14 of the 336 patients (4.2%) had a positive TSPOT using an ELISPOT reader (ER) (Table 2). Only 3 patients had a positive result for more than 1 diagnostic test, and only 1 patient had positive test results for all 3 diagnostic tests (TST, QFT-3G, and TSPOT).

**Table 2:** Prevalence of a Positive Diagnostic Test for Latent Tuberculosis Infection

Diagnostic test	Positive test result (Percentage Positive)
**TST**	7 (2.5%)

**QFT-3G**	9 (2.7%)

**TSPOT**	14 (4.2%)

**At least 1 positive test**	27 (8.0%)

**All 3 tests positive**	1 (0.3%)

TST = Tuberculin Skin Test

QFT-3G = QuantiFERON-TB Gold in Tube

TSPOT = T-SPOT. TB

### Tuberculin Skin Test (TST)

TST results were available for 278 (82.7%) persons. Seven patients (2.5%) had a positive TST with a mean induration of 16 mm (range 11–25 mm). All patients with a positive TST had a CD4 count ≥ 200 cells/μl. There was no difference in the mean CD4 count of patients with a positive skin test as compared to a negative skin test (448 cells/μl versus 369 cells/μl, p = 0.5).

### Interferon-γ Release Assays (IGRAs): QuantiFERON-TB Gold In Tube (QFT-3G) and T-SPOT.TB (TSPOT)

#### QFT-3G

Nine patients (2.7%) had a positive QFT-3G, 321 patients (95.5%) had a negative result, and 6 patients (1.8%) had an indeterminate test result. The median amount of interferon-γ produced in response to the mitogen was > 10 IU/ml (range 0.2–> 10 IU/ml). The median amount of interferon-γ produced in response to TB specific antigens was 0.03 IU/ml (range 0–> 10 IU/ml). Among patients with a positive QFT-3G test result; the median interferon-γ response to TB specific antigens was 0.7 IU/ml (range 0.4–3.5 IU/ml).

There was no difference between the mean CD4 counts of patients with a positive versus a negative QFT-3G (441 cells/μl and 367 cells/μl, p = 0.3). There was a significant correlation between the CD4 count and interferon-γ response to mitogen. (ρ = 0.44, p < 0.001). There was no significant correlation between CD4 count and interferon-γ response to TB specific antigens (ρ = 0.1, p = 0.08). There was no association between the degree of induration with TST and the interferon-γ response to TB specific antigens (ρ = 0.08, p = 0.2). In univariate analysis the only factor associated with a positive QFT-3G was a past history of malignancy (OR = 6.8, 95% CI 1.3, 36.4).

#### TSPOT

Fourteen persons (4.2%) had a positive TSPOT using an ELISPOT reader (ER), 275 patients (81.8%) had a negative test, and 47 patients (14.0%) had an indeterminate test result. There was no difference in CD4 counts among patients with a positive compared to those with a negative TSPOT test (303/μl versus 372/μl, p = 0.3). The mean number of spots for panel A (ESAT-6) was 3 spot forming cells, (range 0–167) and for panel B (CFP 10) was 2.1 spot forming cells (range 0–660).

There was no correlation between the CD4 count and interferon-γ response to mitogen (ρ = 0.01, p = 0.8) or interferon-γ response to ESAT-6 or CFP 10 (ρ = 0.02, p = 0.8 and ρ = 0.1, p = 0.07 respectively). However, there was a significant association between the degree of induration with TST and the interferon-γ response to ESAT-6 and CFP-10 (ρ = 0.16, p = 0.007). In univariate analysis and multivariate analysis, a positive TSPOT was associated with a prior history of LTBI (univariate OR = 10.5, 95% CI 1.9, 59.9, multivariate OR = 10, 95% CI 1.7, 59.7).

The number of persons with a positive TSPOT and an indeterminate test differed when using the ELISPOT Reader compared to visual readings with a magnifying glass. There was moderate concordance between ER and visual inspection [κ = 0.58 (95% CI 0.47, 0.68)] (Table 3). Results from the ER were used in assessing prevalence and risk factors for a positive test and concordance with the other diagnostic tests.

**Table 3:** Comparison of TSPOT results by ELISPOT reader (ER) vs Visual Exam (VE).

	Positive by ER (n = 14)	Negative by ER	Invalid by ER
**Positive by VE** **(n = 31)**	13	17	1

**Negative by VE**	0	244	16

**Invalid by VE**	1	14	30

κ = 0.58, 95% CI (0.47, 0.68)

### Concordance between Diagnostic Tests

A total of 3 patients had a positive result for more than 1 diagnostic test and 1 patient tested positive for TST, QFT-3G, and TSPOT. There was poor concordance between TST and QFT-3G [κ = 0.23, (95% CI -0.05, 0.51)] (Table 4), between TSPOT and TST [κ = 0.16, (95% CI -0.06, 0.39)] (Table 5), and between the two IGRAs, QFT-3G vs TSPOT [κ = 0.06, 95% CI (-0.1, 0.2)] (Table 6). In univariate analysis, TST-positive/QFT-3G-negative discordance was associated with a history of BCG vaccination (OR = 8.0, 95% CI 1.3, 50.7) and a prior history of LTBI (OR = 16.0, 95% CI 1.4, 177). TST-positive/TSPOT-negative discordance was associated with BCG vaccination (OR 17.7, 95% CI 1.9, 167). TSPOT-positive/QFT-3G-negative discordance was associated with a prior history of LTBI (OR = 13.7, 95% CI 2.0, 93.6). No risk factors were found to be associated with QFT-3G-positive/TST-negative, QFT-3G-positive/TSPOT-negative, or TSPOT-positive/TST-negative discordance.

**Table 4:** Degree of Concordance among Three Diagnostic Tests for Latent Tuberculosis Infection Concordance between TST and QFT-3G

	QFT-3G positive (n = 9)	QFT-3G negative	QFT-3G indeterminate
**Positive TST (n = 7)** **(> 5 mm induration)**	2	5	0

**Negative TST**	7	259	5

**TST not read**	0	58	0

κ = 0.23, 95% CI (-0.05, 0.51).

**Table 5:** Concordance between TST and TSPOT

	TSPOT positive (n = 14)	TSPOT negative	TSPOT invalid
**Positive TST** **(n = 7)**	2	4	1

**Negative TST**	10	219	42

**TST not read**	2	52	4

κ = 0.16, 95% CI (-0.06, 0.39).

**Table 6:** Concordance between QFT-3G and TSPOT

	TSPOT positive (n = 14)	TSPOT negative	TSPOT invalid
**QFT-3G positive** **(n = 9)**	1	6	2

**QFT-3G negative**	13	264	44

**QFT-3G indeterminate**	0	4	1

κ = 0.06, 95% CI (-0.1, 0.2).

TST = Tuberculin Skin Test

QFT-3G = QuantiFERON-TB Gold in Tube

TSPOT = T-SPOT.TB

### Impact of Varying Cutoff Values on Concordance

A QFT-3G test is considered positive if the interferon-γ is ≥ 0.35 IU/ml based on previously published data and the manufacturer's recommendations [9]. Lowering the cutoff value for a positive QFT-3G to ≥ 0.05, ≥ 0.15 and ≥ 0.25 IU/ml did not impact concordance between tests. The cutoff for a positive TSPOT test is ≥ 6 spot forming cells, decreasing the cutoff value of a positive TSPOT to ≥ 5 spots, ≥ 4 spots, ≥ 3 spots did not improve concordance between tests. We evaluated the impact of CD4 counts on concordance by measuring κ in patients with CD4 counts ≥ 200/μl, ≥ 300/μl, and ≥ 400/μl. There was a slight increase in κ values at higher CD4 counts, but concordance remained poor.

### Risk Factors for an Indeterminate TSPOT or QFT-3G Test Result

An indeterminate test result occurred in 6 patients (1.8%) with QFT-3G. Five occurred due to an inadequate mitogen response and one occurred due to high background levels of interferon-γ in the negative control. In univariate analysis, there was no significant association between an indeterminate QFT-3G and a CD4 count ≤ 200/μl (OR = 4.8, 95% CI 0.9, 26.8).

An indeterminate TSPOT result occurred in 47 patients (14%). Twenty-eight patients had an inadequate interferon-γ response to the mitogen, 14 had insufficient cells, and 5 patients had high background levels of interferon-γ in the negative control. In multivariate analysis, for TSPOT, a CD4 count ≤ 200/μl was associated with an increased risk of an indeterminate test result (OR = 3.4, 95% CI 1.8, 6.5). We then pooled data for the indeterminate QFT-3G and indeterminate TSPOT and found that a CD4 count ≤ 200/μl was associated with an indeterminate IGRA test (OR = 3.6, 95% 1.9, 6.8) (Table 7).

**Table 7:** Multivariate analysis of risk factors associated with an Invalid TSPOT or Indeterminate QFT-3G (n = 51)

Risk factor	Odds Ratio and 95% Confidence Interval
Age > 40 years	0.9 (0.5, 1.6)

African-American Race	0.8 (0.3, 1.7)

Male gender	0.5 (0.2, 1.0)

**CD4 count ≤ 200 cells/μl**	**3.6 (1.9, 6.8)**

QFT-3G = QuantiFERON-TB Gold in Tube

TSPOT = T-SPOT. TB

## Discussion

To our knowledge this is the largest study to date to look at TST, TSPOT and QFT-3G in HIV seropositive persons. The most striking finding in our study was the poor concordance found between the three different tests with κ values ranging from 0.06–0.23. The overall prevalence of a positive test with TST was 2.5%, by QFT-3G was 2.7% and TSPOT was 4.2%. However only three people tested positive with more than one test and only one person tested positive for all three tests. Other studies from countries with a low prevalence of TB have also found poor concordance between TST and QFT-3G [15,18,19]. However no other study has looked at concordance between TST and TSPOT in low prevalence countries. In studies from Africa concordance between TST and IGRAs has been fair [13,20].

We evaluated whether lowering the interferon-γ cutoff would improve concordance between tests since the current cutoff values are based on receiver operator characteristics in immunocompetent individuals and it is known that HIV-infected patients have impaired interferon responses [21]. Lowering the interferon cutoff did not improve concordance. Studies have shown that TST is more reliable at CD4 counts ≥ 100/μl [22]. We wanted to define a similar CD4 cutoff for IGRAs. However, limiting analysis to patients with higher CD4 counts did not improve concordance in our study population.

Another finding from our study was that TSPOT test gave the largest number of positive results which has also been confirmed in other studies [20]. On univariate analysis we found a prior history of LTBI was associated with a positive TSPOT and with TSPOT positive/QFT-3G negative discordance suggesting that perhaps TSPOT is a more sensitive test. However these results need to be confirmed with multivariate analysis in a larger study. A meta-analysis showed that TSPOT was more sensitive than QFT-3G at diagnosing active TB [11]. Contact investigation studies in both immunocompetent and immunocompromised hosts have shown that TSPOT is more likely to be positive than TST [23-25] and QFT-3G [26].

Our study is the first to compare the results from TSPOT read with a magnifying lens and those with an ELISPOT reader and found moderate agreement between the tests. However if we had relied on only the magnifying lens we would have had 31 tests positive as opposed to 14. We feel results are likely to be more accurate when interpreted using an ELISPOT reader. If these tests do become available in developing countries the cost of the ELISPOT Reader will need to be considered when deciding what test to use.

Another issue that arises in using IGRAs in immunocompromised persons is the rate of indeterminate test results. In our study an indeterminate test result occurred in 14% of patients with TSPOT and 1.8% of patients with QFT-3G. The high rate of indeterminate TSPOT tests likely occurred due to inadequate CD4 cells. Other studies in HIV seropositive individuals have also found that around 10% of patients have an indeterminate result [20]. In our study an indeterminate result was associated with a CD4 count of < 200 cells/μl. Patients with indeterminate results will require retesting, testing with a larger volume of blood (in order to obtain more PBMCs for TSPOT), or use of a different diagnostic test. It is unclear therefore how useful these tests will be in severely immunocompromised patients.

Our study is subject to several limitations. The low prevalence of positive test results limited our ability to evaluate risk factors associated with positive test results and factors associated with discordance. Studies in countries with a higher prevalence of LTBI may help overcome this problem. In addition, the lack of a gold standard makes it difficult to determine which diagnostic test is more sensitive or specific for LTBI.

Given the results of our study and the limited data currently available in the literature, it is unclear if IGRAs can be used alone for diagnosis of LTBI in HIV-infected individuals. Some authors have suggested a combined approach of TST and IGRA [15]. We feel this would lead to confusion for physicians when there are discordant results. Questions that remain are whether in high-incidence countries there is better concordance between IGRAs and TST; can we define a lower interferon-γ cutoff value for patients who are immunocompromised or have HIV; is there a CD4 cutoff value below which these tests are no longer useful; and is IGRA positivity an accurate predictor of progression to active TB.

## Conclusion

In conclusion, we report the largest study to date to assess the use of IGRAs in HIV-infected persons. Our findings indicate a low prevalence of LTBI, poor concordance between the TST and IGRAs and high rates of indeterminate test results in patients with CD4 counts ≤ 200 cells/μl. Additional studies, preferably with a longitudinal design in HIV-infected individuals and other immunocompromised patients in various clinical settings, are needed to determine the true role IGRAs will play in the diagnosis of LTBI in such patients.

## Abbreviations

LTBI: latent tuberculosis infection; TSPOT: T-SPOT.TB; QFT-3G: Third Generation QuantiFERON-TB Gold in Tube; TST: tuberculin skin test; HIV: human immunodeficiency virus; TB: tuberculosis; BCG: Bacillus Calmette Guerin; IGRA: Interferon Gamma Release Assays; ESAT6: early secretory antigen6; CFP10: culture filtrate protein 10; PPD: purified protein derivative; CDC: Centers for Disease Control; FDA: Food and Drug Administration; ER: Elispot reader; ELISA: Enzyme Linked Immunosorbent Assay; OR: odds ratio; CI: confidence interval; PBMC: peripheral blood mononuclear cells.

## Competing interests

The authors declare that they have no competing interests.

## Authors' contributions

NT was responsible for data analysis and drafting and revising the manuscript. US contributed to data analysis, interpretation and writing the methods and results section. BH performed all the interferon-γ release assays (QuantiFERON-TB Gold in Tube and TSPOT.TB assays) and contributed to the methods section of the paper. AA was responsible for data collection and entry and contributed to the methods section of the paper. HMB, JT, PW and RA were responsible for the conception and study design and also contributed to the discussion section and editing the entire manuscript. HMB also provided oversight for the entire project and revised the manuscript. All authors read and approved the final manuscript.

## Financial support

Supported in part by Centers for Disease Control and Prevention (CDC) Tuberculosis Epidemiologic Studies Consortium (contract number 200-2001-00086), the CDC Foundation, and the Emory Center for AIDS Research (P30 AI050409).

## Disclaimer

The findings and conclusions in this paper are those of the authors and do not necessarily represent the views of the Center for Disease Control and Prevention.

## Pre-publication history

The pre-publication history for this paper can be accessed here:

http://www.biomedcentral.com/1471-2334/9/15/prepub
